# Subcellular Metabolite and Lipid Analysis of *Xenopus laevis* Eggs by LAESI Mass Spectrometry

**DOI:** 10.1371/journal.pone.0115173

**Published:** 2014-12-15

**Authors:** Bindesh Shrestha, Prabhakar Sripadi, Brent R. Reschke, Holly D. Henderson, Matthew J. Powell, Sally A. Moody, Akos Vertes

**Affiliations:** 1 Department of Chemistry, W. M. Keck Institute for Proteomics Technology and Applications, The George Washington University, Washington, D.C., United States of America; 2 Department of Anatomy and Regenerative Biology, The George Washington University, Washington, D.C., United States of America; 3 Protea Biosciences, Morgantown, West Virginia, United States of America; Institut de Génétique et Développement de Rennes, France

## Abstract

*Xenopus laevis* eggs are used as a biological model system for studying fertilization and early embryonic development in vertebrates. Most methods used for their molecular analysis require elaborate sample preparation including separate protocols for the water soluble and lipid components. In this study, laser ablation electrospray ionization (LAESI), an ambient ionization technique, was used for direct mass spectrometric analysis of *X. laevis* eggs and early stage embryos up to five cleavage cycles. Single unfertilized and fertilized eggs, their animal and vegetal poles, and embryos through the 32-cell stage were analyzed. Fifty two small metabolite ions, including glutathione, GABA and amino acids, as well as numerous lipids including 14 fatty acids, 13 lysophosphatidylcholines, 36 phosphatidylcholines and 29 triacylglycerols were putatively identified. Additionally, some proteins, for example thymosin β4 (Xen), were also detected. On the subcellular level, the lipid profiles were found to differ between the animal and vegetal poles of the eggs. Radial profiling revealed profound compositional differences between the jelly coat vitelline/plasma membrane and egg cytoplasm. Changes in the metabolic profile of the egg following fertilization, e.g., the decline of polyamine content with the development of the embryo were observed using LAESI-MS. This approach enables the exploration of metabolic and lipid changes during the early stages of embryogenesis.

## Introduction

The early stages of embryonic development following fertilization in animals are characterized by synchronous cell divisions, the onset of transcription of genes that will pattern the embryo, and local signaling events that transition a ball of equipotent cells into regions that express different tissue fates [1], [2]. These events are common across different species of animals, but can occur for different lengths of times and at different stages of morphogenesis.

The African clawed frog, *Xenopus laevis*, has been used extensively to study the cellular and molecular events of early embryogenesis. This species is used because they are easily bred in the laboratory, are disease resistant, have a reproductive response that is independent of season, and produce a large number of eggs per spawning. In particular, the large size of the oocytes, ova (unfertilized eggs) and embryos make them easy to manipulate and analyze by standard biochemical and molecular biology approaches [3], [4], [5], [6], [7].

*X. laevis* oocytes and eggs have been an important source of material for elucidating the molecular regulation of the cell cycle. For example, traditional protein chemistry approaches led to the identification of maturation promoting factor and the cyclin proteins [8], [9]. More recently, the oocyte has been used to identify the proteins involved in nuclear transport and DNA replication [6]. To reveal the biochemical makeup, elucidate low abundance proteins, and study the kinetics of developmental and cellular events, *X. laevis* oocytes have been analyzed by high performance liquid chromatography (HPLC) [10], [11] [12], time-of-flight (TOF) secondary ion mass spectrometry (SIMS) [13], gas chromatography (GC) mass spectrometry (MS) [14], 2D gel electrophoresis followed by electrospray ionization (ESI) MS [15], and liquid chromatography-tandem MS (LC-MS/MS) [2]. To study the types and amounts of lipids extracted from *Xenopus* eggs, HPLC methods were developed for the detection of multiple species [10], [12]. Similarly, with an HPLC method for characterizing the carbohydrates in the jelly coat of *Xenopus* eggs, specific oligosaccharides were identified and assigned to the three specific layers of the coat [11], [16]. In another study, LC-MS/MS was used to monitor the amounts of alanine in the early embryo, resulting in a new hypothesis for the increase in cell cycle time at the mid-blastula transition [2]. Using a GC-MS method for analyzing metabolites from volume-limited samples, e.g., *X. laevis* eggs, good reproducibility was shown for volumes as small as 10 nL. However, the method required silylation of some metabolites to increase their volatility for analysis using GC [14]. On a subcellular scale, 3D molecular images of freeze dried *Xenopus* oocytes were obtained under optimized sample preparation conditions using TOF-SIMS [13]. While all of these methods for *X. laevis* oocytes and embryo analysis have been shown to capture important aspects of its biochemistry, most of them require elaborate sample preparation prior to analysis that might alter subcellular localization of important developmental molecules.

An advantage of direct analysis methods is that they can report on the unperturbed composition of biological specimens, ideally in their living, natural state. Recently, the analysis of single oocytes and embryos of mice and bovine species by desorption electrospray ionization (DESI) mass spectrometry provided new insight into lipid metabolism changes during early embryonic development [17], [18].

In another direct analysis technique, laser ablation electrospray ionization (LAESI) MS, the energy from a focused mid-infrared laser pulse is absorbed by the water molecules in a cell or tissue leading to the ablation sampling of adjacent cellular content [19]. Each consecutive laser pulse ablates a successive layer of the sample enabling analysis at a defined subcellular depth or of an entire cell [20], [21]. Combination of LAESI with microdissection of plant cells reveals subcellular metabolite gradients between the cytoplasm and the nucleus [22]. Diverse applications of LAESI-MS include the analysis of virally infected immune cells as well as comparative analysis of plant cell phenotypes [23], [24]. Because the developing egg is a spatially and temporally dynamic system that is sensitive to environmental and thus preparatory changes, we used the direct LAESI-MS analysis of individual *X. laevis* eggs and embryos to explore subcellular asymmetries and potential changes during significant developmental transitions. Using LAESI-MS to probe these specimens required minimal sample preparation, was performed at atmospheric pressure, and each ablation event occurred instantaneously (in less than a second) on the developmental time scale. The analyses were performed on living eggs and embryos that were minimally perturbed from their natural state.

Some of the metabolic profiles of the unfertilized egg obtained from the LAESI-MS analyses were compared to results reported previously in the literature and showed good agreement, thus validating the approach. In addition we identified subtle differences between the unfertilized egg, in which the first meiotic division is completed, and the fertilized egg and early cleavage stage embryos. Importantly, we found differences between the animal and vegetal poles of the egg, which are predisposed to different developmental fates. Since these early developmental events are shared across animals, a direct analysis of the cellular constituents should provide fundamental information about the common biochemical processes that regulate them. This simple approach using a single, live embryo in its natural state should be ideal for analyses of other embryos that are difficult to collect and are small in size.

## Materials and Methods

### Ova and Embryos

*X. laevis* eggs and sperm were obtained from mature animals kept in 30 gallon aquaria at 21°C in natural daylight and fed frog brittle 2 times per week. Oocyte maturation was induced by injecting frogs with 1000 U human chorionic gonadotropin (Sigma) 12 hours prior to removal of eggs as described elsewhere [25]. Mature eggs were gently squeezed from female frogs into Petri dishes; some were collected prior to fertilization and others were fertilized in vitro and collected at various stages after sperm entry. Sperm were obtained from minced testes of sacrificed males that were anesthetized by submersion in an ice bath with tricaine methanesulfonate. Eggs were fertilized *in vitro* by adding the released sperm to the eggs and adding 0.1× MBS (1× = 88 mM NaCl, 1 mM KCl, 1 mM MgSO_4_, 0.7 mM CaCl_2_, 5 mM HEPES, pH 7.8 and 2.5 mM NaHCO_3_) [26]. The jelly coat was removed from some samples by washing the eggs with 2% cysteine solution (pH 8.0, adjusted by 10 M NaOH) for 4 minutes followed by washing in 0.1× Steinberg's solution (1× = 60 mM NaCl, 0.67 mM KCl, 0.83 mM MgSO_4_, 0.34 mM Ca(NO_3_)_2_, 4 mM Tris-HCl, 0.66 mM Tris Base and pH 7.4). For some experiments the vitelline membrane was manually removed from each egg using sharpened forceps. Unfertilized eggs of *X. laevis* were also purchased from Xenopus Express, Inc. (Brooksville, FL) for some confirmatory studies. All animals use in these experiments followed the U.S. Public Health Service Policy of Humane Care and Use of Laboratory Animals and were approved by the George Washington University (GWU Animal Study Protocol: #A-3205-01; A237).

### Chemicals

All chemicals for egg preparation were reagent grade and obtained from Sigma. For the electrospray solution, highly purified spectral-grade methanol and water solvents were obtained from Acros Organics (Geel, Belgium), and glacial acetic acid was purchased from Fluka (Munich, Germany). All of the chemicals were used without further purification.

### LAESI-MS

The LAESI ion source used was similar to those described earlier [19]. Briefly, mid-infrared laser pulses at 2940 nm wavelength and 20 Hz repetition rate were produced by a Nd:YAG laser driven optical parametric oscillator system (Opolette 100, Opotek, Carlsbad, CA). Laser pulses with an average 320 µJ/pulse energy were focused to about 200–300 µm diameter spot with either a 150 mm focal length CaF_2_ lens or a 75 mm ZnSe lens. The eggs/embryos were placed on a pre-cleaned microscope glass slide held 15 mm below the electrospray axis that was in line with the mass spectrometer orifice. In the home-built electrospray system, a syringe pump supplied 50% methanol solution containing 0.1% acetic acid through a tapered tip metal emitter (length 5 cm, tube OD 320 µm and tip ID 50 µm) at a flow rate of 300–400 nL/min. The electrospray emitter was held at 2800–3300 V. Positive ions were collected by either an orthogonal acceleration TOF mass spectrometer (Q-TOF Premier, Waters Co., Milford, MA) with a mass range of *m/z* 50–2000 and a typical resolution of 8,000 (FWHM) or with a high performance TOF mass spectrometer (Synapt G2S, Waters Co., Milford, MA) with a mass range of *m/z* 50–2000 and a typical resolution of 30,000 (FWHM) [27].

### Data Analysis

The electrospray background ion signal was subtracted from the collected LAESI mass spectra in the MassLynx 4.1 software (Waters Co., Milford, MA). Putative peak assignments for metabolites and lipids were based on accurate mass measurements, isotope distribution patterns, database searches, data mining of the related literature, and in some cases tandem MS analysis. Multiple databases were used for the metabolite and lipid searches, including METLIN [28], MetaCyc [29], Lipid Maps [30], HMDB [31] and KEGG [32], [33]. They were last accessed on August 12, 2014. The NIST Isotope Calculator program (ISOFORM, Version 1.02) was used to calculate monoisotopic masses.

## Results and Discussions

### Ovum Analysis

**Figure 1:**
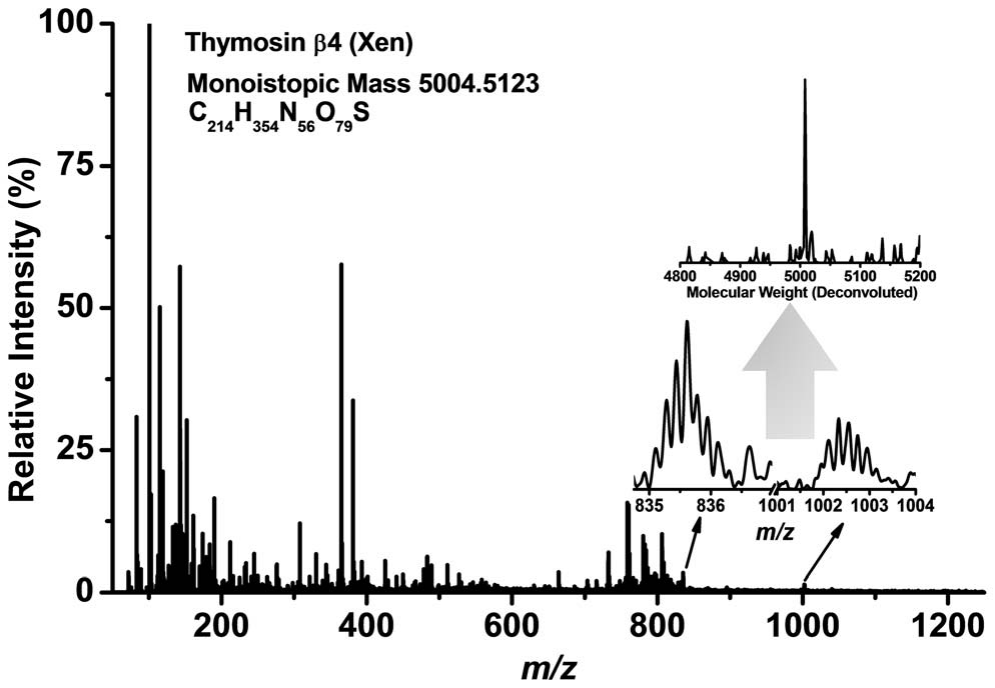
A typical positive ion LAESI mass spectrum of a single unfertilized *X. laevis* egg is dominated by numerous metabolite and lipid peaks. Protonated thymosin β4 (Xen) peptide in the 6+ and 5+ charge states (*m/z* 835 and 1002, respectively) is also present in the spectrum. Deconvolution of the peptide peaks, shown in the inset, yields a mass of 5004 Da corresponding to the molecular mass of thymosin β4 (Xen).

A single *X. laevis* egg is ∼1.4 mm in diameter and has an approximate volume of 400 nL. A typical LAESI mass spectrum from a single unfertilized *X. laevis* egg without the removal of its jelly coat is shown in Fig. 1. Most of the ions below *m/z* 450 correspond to small metabolites, whereas many ions between *m/z* 450 and 900 are assigned to lipids. For a typical unfertilized *X. laevis* ovum, putative assignments of the ions generally found in the *m/z* <550 region reveals 52 small metabolites (see Table 1). Many of these metabolites, e.g., amino acids, organic acids, and redox buffering agents, fulfill an essential role in cell development and serve as building blocks for cellular biosynthesis. Using liquid extraction and LC-MS/MS, a previous study followed 48 metabolites in *X. laevis* embryos [2]. In Table 1, sixteen metabolites found by both the LC-MS/MS and LAESI-MS methods are marked by asterisks. The advantage of LAESI-MS over conventional techniques is that it rapidly (within seconds) identifies metabolites in a single egg with minimum perturbation before analysis. It also provides complementary data for improved coverage of the small metabolites.

**Table 1:** Putative identification of metabolite ions from LAESI-MS of an unfertilized *X. laevis* egg.

Metabolite	Chemical formula	Monoisotopic mass	Measured mass	Δm (mDa)
carbamate	CH_2_NO_2_	82.998 (Na+)	82.997	−1
putrescine	C_4_H_12_N_2_	89.108 (H^+^)	89.106	−2
alanine*	C_3_H_7_NO_2_	90.056 (H^+^)	90.054	−2
choline*	C_5_H_13_NO	104.108 (H^+^)	104.106	−1
propionic acid	C_3_H_6_O_2_	113.001 (K^+^)	113.012	11
creatinine	C_4_N_3_H_7_O	114.067 (H^+^)	114.064	−3
propanediol	C_3_H_8_O_2_	115.016 (H^+^)	115.022	6
proline*	C_5_H_9_NO_2_	116.071 (H^+^)	116.071	0
		138.053 (Na^+^)	138.053	0
niacinamide	C_6_H_6_N_2_O	123.056 (H^+^)	123.049	−7
hydroxyethylphosphonate	C_2_H_7_O_4_P	127.016 (H^+^)	127.023	7
butyric acid	C_4_H_8_O_2_	127.016 (K^+^)	127.023	7
octenol	C_8_H_16_O	129.128 (H^+^)	129.135	7
creatine	C_4_H_9_N_3_O_2_	132.077 (H^+^)	132.075	−2
imidazole acetaldehyde (or)	C_5_H_6_N_2_O	133.038 (Na^+^)	133.033	−5
N-acetylimidazole	C_5_H_6_N_2_O	133.038 (Na^+^)	133.033	−5
malic acid*	C_4_H_6_O_5_	135.029 (H^+^)	135.030	1
adenine	C_5_H_5_N_5_	136.062 (H^+^)	136.053	−9
threonic acid or erythronic acid	C_4_H_8_O_5_	137.045 (H^+^)	137.044	−1
hexenal	C_6_H_10_O	137.037 (K^+^)	137.044	7
valeric acid or pentanoic acid	C_5_H_10_O_2_	141.032 (K^+^)	141.038	6
gamma aminobutyric acid amide	C_4_H_10_N_2_O	141.043 (K^+^)	141.038	−5
gamma-hydroxybutyric acid	C_4_H_8_O_3_	143.011 (K^+^)	143.018	7
spermidine	C_7_H_19_N_3_	146.166 (H^+^)	146.164	−2
guaiacol	C_7_H_8_O_2_	147.042 (Na^+^)	147.048	6
glutamate*	C_5_H_9_NO_4_	148.061 (H^+^)	148.058	−3
methionine*	C_5_H_11_NO_2_S	150.059 (H^+^)	150.047	12
guanine* or hydroxyadenine	C_5_H_5_N_5_O	152.057 (H^+^)	152.054	−3
		174.039 (Na^+^)	174.038	−1
		190.013 (K^+^)	190.012	−1
dihydroorotic acid	C_5_H_6_N_2_O_4_	159.041 (H^+^)	159.040	−0
homospermidine	C_8_H_21_N_3_	160.181 (H^+^)	160.180	−1
urocanic acid	C_6_H_6_N_2_O_2_	161.033 (Na^+^)	161.029	−4
carnitine*	C_7_H_15_NO_3_	162.113	162.102	11
aspartic acid*	C_4_H_7_NO_4_	172.001 (K^+^)	172.008	7
glycerol 3-phosphate*	C_3_H_9_O_6_P	173.022 (H^+^)	173.028	6
arginine	C_6_H_14_N_4_O_2_	175.120 (H^+^)	175.117	−3
cys gly	C_5_H_10_N_2_O_3_S_1_	179.049 (H^+^)	179.046	−3
phosphocholine	C_5_H_14_NO_4_P	184.074 (H^+^)	184.070	−4
		206.056 (Na^+^)	206.050	−6
histidine*	C_6_H_9_N_3_O_2_	194.033 (H^+^)	194.033	0
phosphocreatine	C_4_H_10_N_3_O_5_P	212.044 (H^+^)	212.042	−2
N-formylmethionine	C_6_H_11_NO_3_S	216.010 (H^+^)	216.010	0
citrate*	C_6_H_8_O_7_	215.0168 (Na^+^)	215.010	−7
dopaquinone	C_9_H_9_NO_4_	234.017 (K^+^)	234.022	5
homocarnosine or balenine	C_10_H_16_N_4_O_3_	241.130 (H^+^)	241.131	1
pantothenate*	C_9_H_17_NO_5_	242.100 (Na^+^)	242.088	−12
		258.074 (K^+^)	258.070	−4
methylthio propylmalic acid	C_8_H_14_O_5_S	245.046 (Na^+^)	245.050	4
		261.020 (K^+^)	261.027	7
acetyldihydrolipoamide	C_10_H_19_NO_2_S_2_	250.094 (H^+^)	250.091	−3
glutathione*	C_10_H_17_N_3_O_6_S	308.092 (H^+^)	308.090	−2
		330.074 (Na^+^)	330.068	−6
		346.048 (K^+^)	346.047	−1
hydroxydesipramine	C_18_H_22_N_2_O	321.137 (K^+^)	321.127	−10
disaccharide or trehalose*	C_12_H_22_O_11_	365.106 (Na^+^)	365.107	1
		381.080 (K^+^)	381.080	0
cholesterol	C_27_H_46_O	369.352 (H^+^, -H_2_O)	369.354	2
adenosine monophosphate (AMP)	C_10_H_14_N_5_O_7_P	386.027 (K^+^)	386.017	−10
adenosine diphosphate (ADP)*	C_10_H_15_N_5_O_10_P_2_	428.037 (H^+^)	428.032	−5
		450.019 (Na^+^)	450.006	−13
		465.993 (K^+^)	465.981	−12
adenosine triphosphate (ATP)*	C_10_H_16_N_5_O_13_P_3_	508.004 (H^+^)	508.002	−2
		529.986 (Na^+^)	529.975	−11

*Metabolites also detected in Ref. [2].

In the mass spectra we noticed the presence of multiply charged peaks at *m/z* 835, 1,002, and 1,251 with charge states of 6+, 5+ (see Fig. 1) and 4+, respectively. Deconvolution of these peaks revealed that they corresponded to thymosin β4 (Xen) (see the inset in Fig. 1). This 44-residue peptide in *Xenopus* has been highly conserved throughout evolution as it only differs from the human thymosin β4 in three amino acid residues [34]. A typical defolliculated oocyte contains 0.5 and 10 picomoles of thymosin β4 (Xen) in stage I and stage VI of the oogenesis, respectively [34]. Our results show that unfertilized eggs, the next step after stage VI oocytes, continue to contain thymosin β4 (Xen) in significant quantities.

### Radial Profiling of Ovum

**Figure 2:**
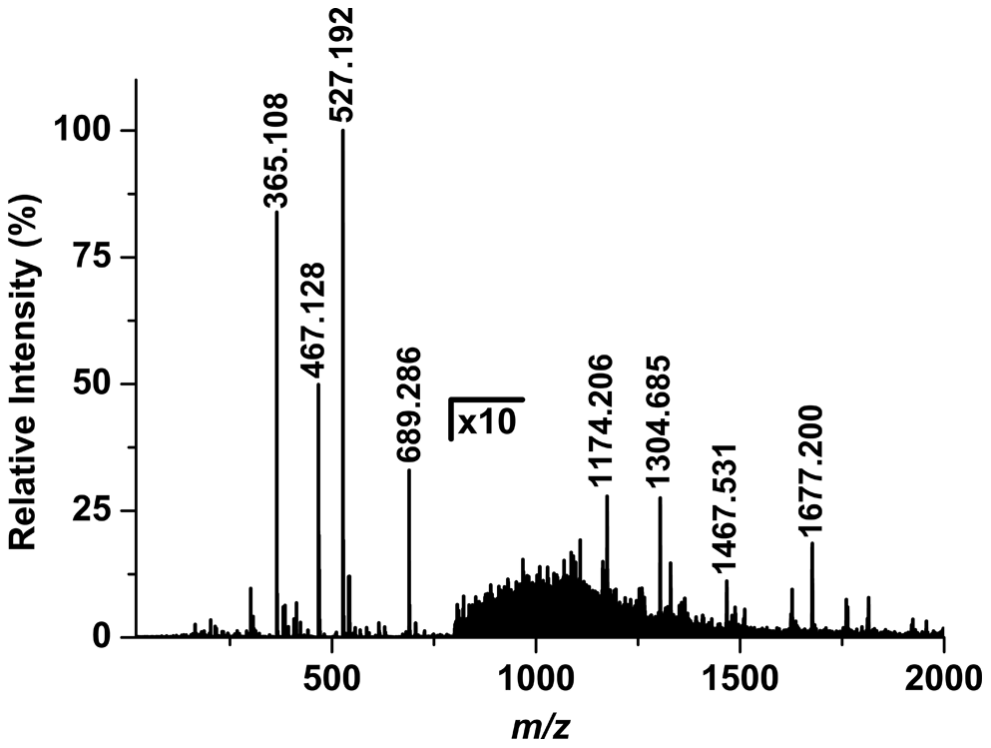
Positive ion LAESI mass spectrum of *X. laevis* egg jelly coat primarily shows sodiated oligosaccharide ions with a set of multiply charged ions at higher *m/z* corresponding to a species with molecular weight of 11728±1 Da.

The *X. laevis* egg is surrounded by a jelly coat that must be penetrated by the sperm during fertilization. The LAESI mass spectra of the jelly coat were dominated by sodiated disaccharide, trisaccharide, and tetrasaccharide molecules at *m/z* 365.108, 527.192 and 689.286, respectively. The *Xenopus* egg jelly is known to contain a 21,073 Da protein, allurin, that serves as a chemoattractant for the sperm [35]. The LAESI mass spectra of the jelly coat did not show the ions for allurin, but exhibited strong signal for multiply charged ions corresponding to a protein with a molecular weight of 11,728±1 Da (see Fig. 2) [36].

**Figure 3:**
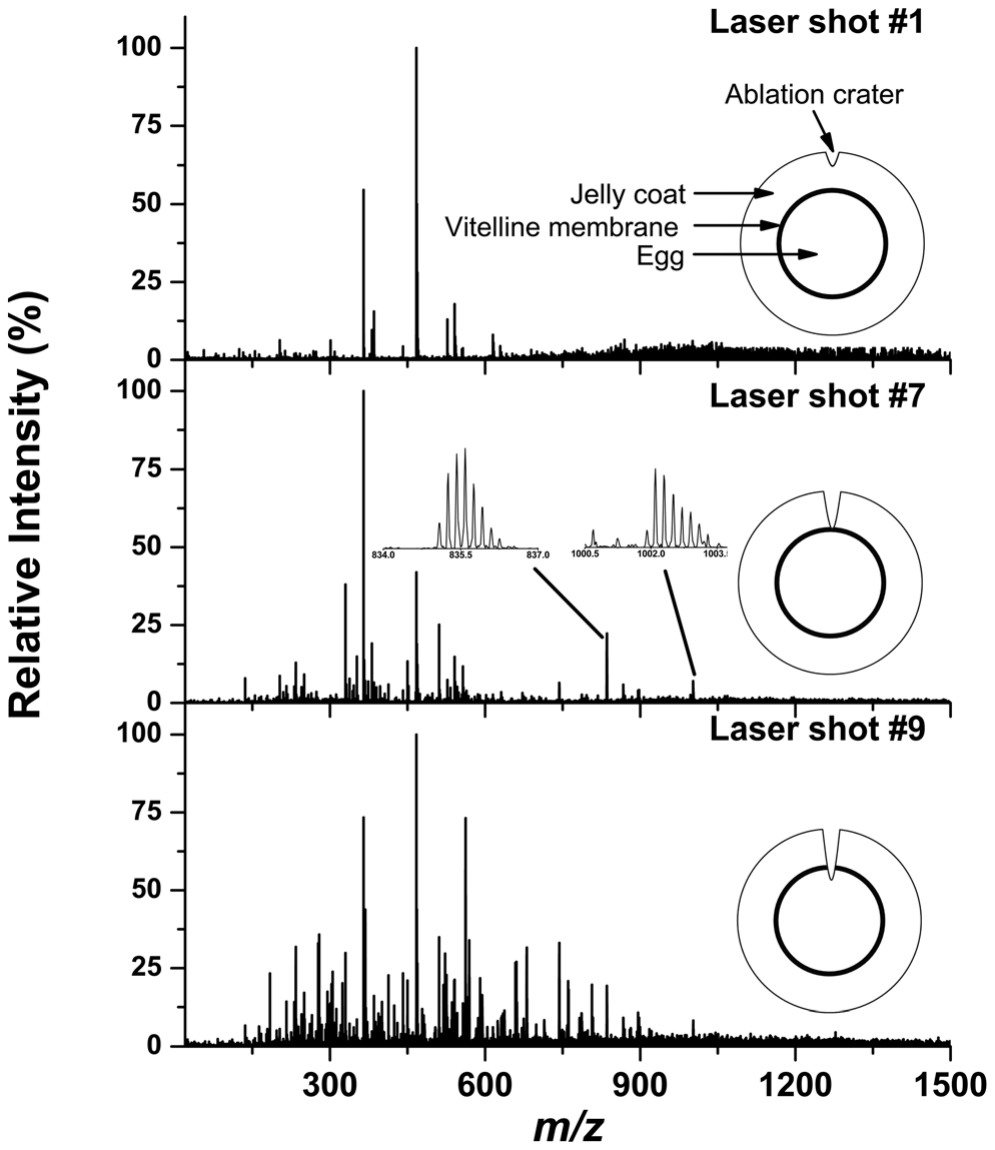
Radial profiling of jellied unfertilized *X. laevis* egg by consecutive laser shots generated mass spectra at each shot corresponding to increasing depths. The first ∼6 laser shots produced mass spectra similar to that of the jelly coat (top spectrum). This was followed by a mass spectrum that showed presence of multiply charged thymosin β4 (Xen) peptide (middle spectrum). After an additional ∼2 laser pulses, other metabolites and lipids characteristic of the cytoplasm were detected (bottom spectrum).

Radial profiling of an unfertilized *X. laevis* egg with a jelly coat was conducted by ablating the same area with multiple laser pulses, producing a mass spectrum for each shot (see Fig. 3). The first approximately six laser shots produced mass spectra similar to that of the jelly coat (see the top panel in Fig. 3). This observation was consistent with the ∼30 µm ablation depth per laser shot and the ∼200 µm thickness of typical jelly coats. Once the jelly coat-related signal diminished, multiply charged thymosin β4 (Xen) peptide ions were detected (see the middle panel in Fig. 3). From its radial localization, it seems that this peptide is associated with the vitelline and/or plasma membranes. After approximately two additional laser shots, metabolites and lipid species characteristic to the egg cytoplasm composition were detected in the mass spectra (see bottom panel in Fig. 3).

### Lipid Composition of Unfertilized Ovum

The cytoplasm of the unfertilized egg contains a high percentage of yolk platelets composed of proteins and lipids for nourishment of the embryonic cells [37]. The major peaks observed between *m/z* 400 and 900 in the LAESI mass spectrum are attributed to lipids. Earlier studies on phospholipid extracts from *X. laevis* eggs showed that the major lipid components were phosphatidylcholines (PC) at ∼20 nmol/egg, followed by phosphatidylethanolamines (PE) at ∼10 nmol/egg, phosphatidylinositols (PI) at ∼4 nmol/egg, lysophosphatidylcholines (LPC) at ∼2 nmol/egg, and phosphatidylserines (PS) at ∼1 nmol/egg [12]. In addition to phospholipids, triacylglycerols (TAG) were the major neutral lipid components found in the *Xenopus* eggs along with fatty acids (FA), cholesterol and diacylglycerols (DAG) [38].

**Figure 4:**
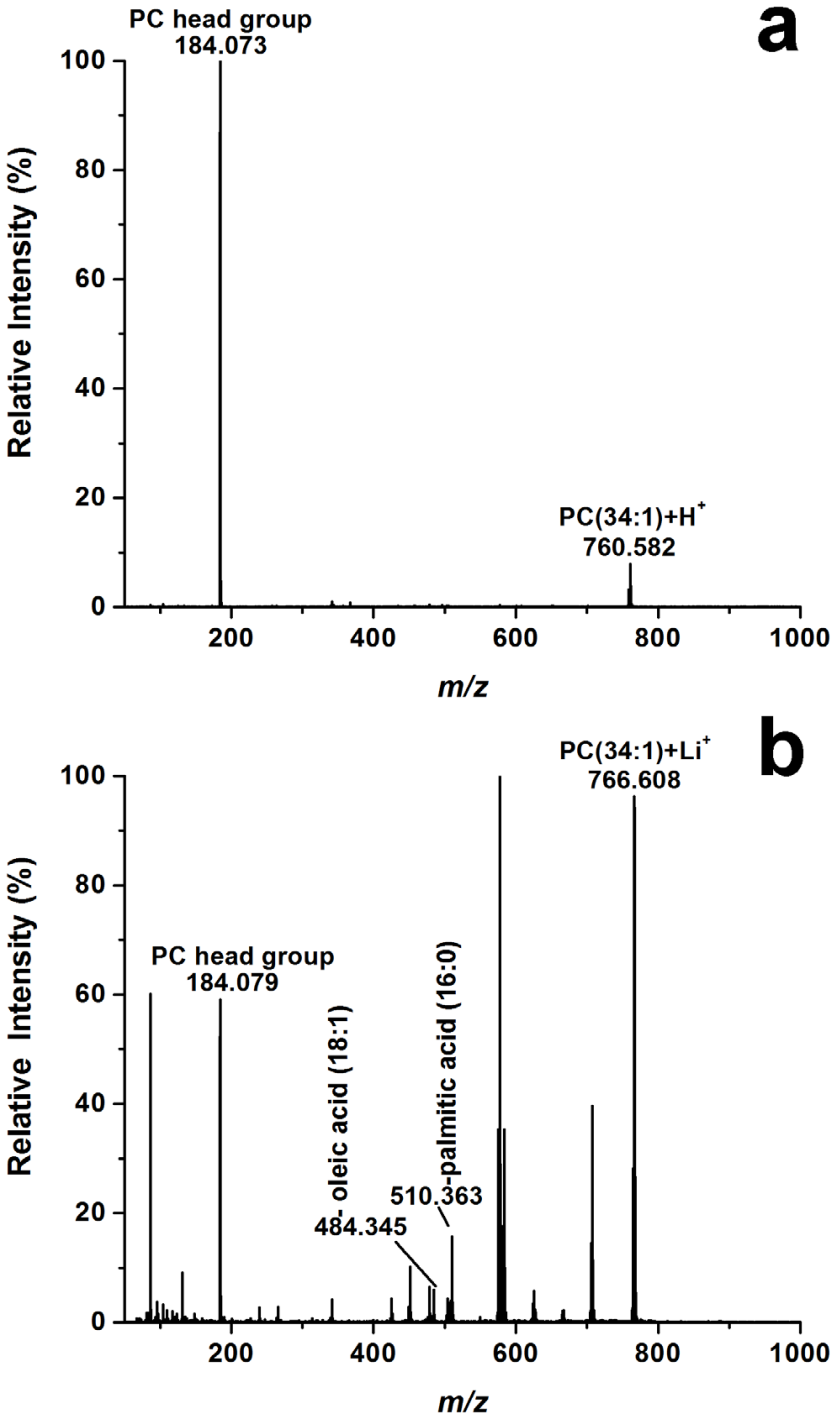
(a) Tandem LAESI mass spectrum of a protonated PC lipid produced a single head group fragment, (b) whereas the tandem MS of its lithiated counterpart produced structure specific fragments enabling the identification of its acyl side chains.

**Figure 5:**
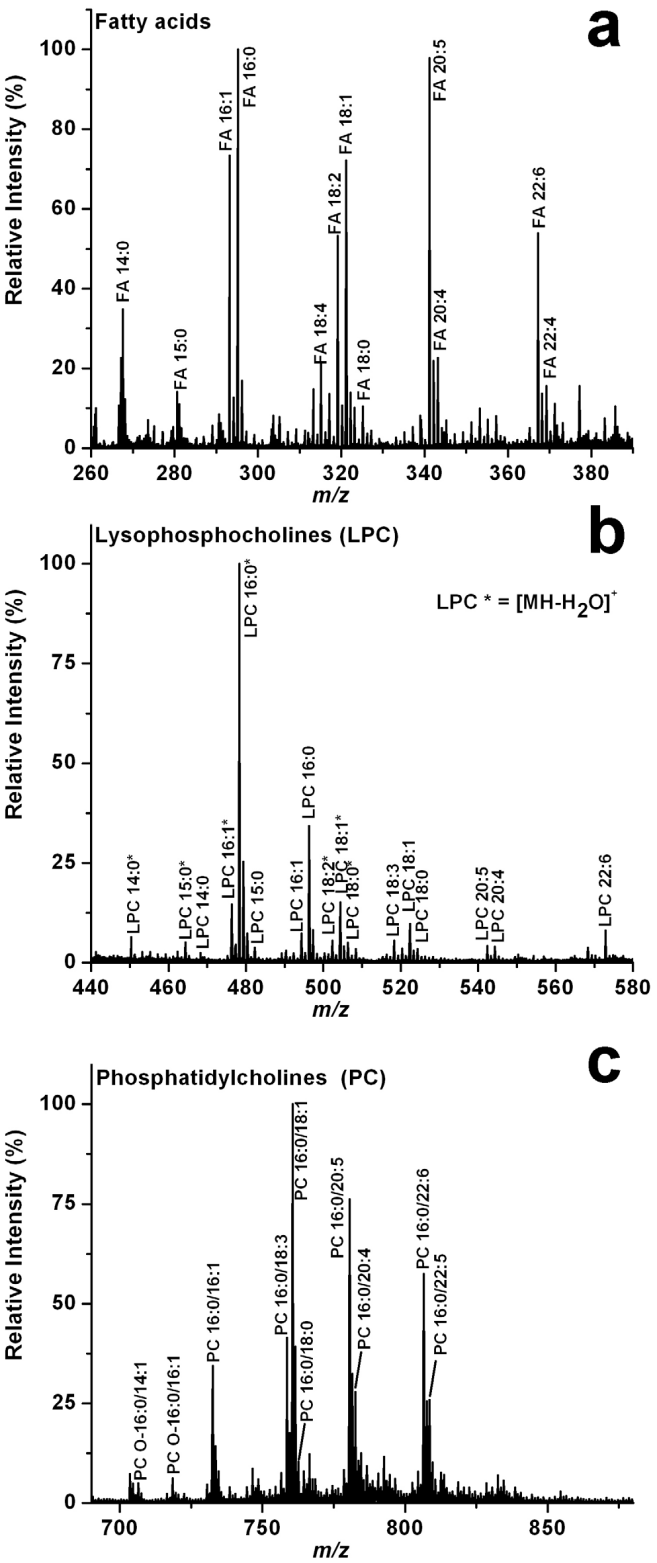
Positive LAESI mass spectra of unfertilized *X. laevis* egg with annotations for selected (a) fatty acids (FA), (b) lysophosphatidylcholines (LPC), and (c) phosphatidylcholines (PC). A complete list of the detected lipid ions is presented in Table 2.

The LAESI mass spectra gave additional insight into the nature of the lipids present in the ovum. A wide variety of lipid classes were identified, including 14 FAs, 13 LPCs, 36 PCs and 29 TAGs. Most of the abundant lipid peaks in these spectra corresponded to PCs, where one of the acyl chains was palmitic acid (16:0), whereas the other acyl chain length and saturation varied between (14:0) and (22:6) (see Table 2). The acyl chain length assignments were based on the tandem mass spectra of the lithiated lipids, [M+Li]^+^, generated by reactive LAESI. In this technique Li^+^ ions from the electrospray solution react with the lipid molecules from the ablated sample [39]. For example, tandem MS of a protonated lipid ion at *m/z* 760.582 produced a single fragment ion corresponding to its phosphocholine head group at *m/z* 184.073 resulting in the overall assignment of PC(34:1) showing only the combined length and number of double bonds for the acyl chains (see Fig. 4a). Further information on the individual acyl chains was obtained by producing and fragmenting the corresponding lithiated species, which showed fragments for the loss of oleic acid (18:1) and palmitic acid (16:0) at *m/z* 510.3631 and 484.3452, respectively (see Fig. 4b). Another major class of lipids in the spectra is LPC that is structurally similar to PC without the acyl group at *sn*-2. Phospholipase A2 is known to degrade PC into LPC and facilitate fertilization. Similar to PC, the major LPC is 16:0, and others include acyl chains between 14:0 and 22:6 (see Table 2). Because LPCs contain a free –OH group on the glycerol moiety, they undergo a loss of H_2_O during protonation and most ions are detected as [MH-H_2_O]^+^. These fragments are often more abundant than the protonated peaks. A few weak peaks corresponding to PE and PS can also be detected in the spectra. The major lipids found in the LAESI experiments and their fatty acid compositions are in good agreement with the literature reports based on extraction, HPLC separation and GC-based fatty acid analysis [12]. A putative list of the assigned lipids observed in the LAESI mass spectra of de-jellied unfertilized *X. laevis* egg is shown in Table 2. LAESI mass spectra with annotations for selected FA, LPC and PC species are shown in Fig. 5.

**Table 2:** Putative peak assignments for fatty acid and lipid ions from LAESI-MS of an unfertilized *X. laevis* egg.

Lipid	Chemical formula	Monoisotopic mass	Measured mass	Δm (mDa)
Propionic acid (3:0)	C_3_H_6_O_2_	113.001 (K^+^)	112.992	−9
Butenoic acid or Crotonic acid (4:1)	C_4_H_6_O_2_	125.001 (K^+^)	124.998	−3
Butyric acid (4:0)	C_4_H_8_O_2_	127.016 (K^+^)	127.010	−6
Pentanoic acid	C_5_H_10_O_2_	141.032 (K^+^)	141.039	7
Caprylic acid (8:0)	C_8_H_16_O_2_	183.079 (K^+^)	183.072	−7
FA(14:0)	C_14_H_28_O_2_	267.173 (K^+^)	267.167	−6
FA(15:0)	C_15_H_30_O_2_	281.188 (K^+^)	281.184	−4
FA(16:1)	C_16_H_30_O_2_	277.214(Na^+^)	277.200	−14
		293.188 (K^+^)	293.179	−9
FA(16:0)	C_16_H_32_O_2_	279.230 (Na^+^)	279.217	−13
		295.204 (K^+^)	295.195	−9
FA(18:5)	C_18_H_26_O_2_	297.183 (Na^+^)	297.194	11
FA(18:4)	C_18_H_28_O_2_	299.199 (Na^+^)	299.186	−13
		315.173 (K^+^)	315.157	−16
FA(18:3)	C_18_H_30_O_2_	317.188 (K^+^)	317.183	−5
FA(18:2)	C_18_H_32_O_2_	303.230 (Na^+^)	303.223	−7
		319.204 (K^+^)	319.195	−9
FA(18:1)	C_18_H_34_O_2_	321.220 (K^+^)	321.214	−6
FA(18:0)	C_18_H_36_O_2_	323.235 (K^+^)	323.226	−9
FA(20:5)	C_20_H_30_O_2_	325.214 (Na^+^)	325.204	−10
		341.188 (K^+^)	341.181	−7
FA(20:4)	C_20_H_32_O_2_	327.230 (Na^+^)	327.222	−8
		343.204 (K^+^)	343.200	−4
FA(22:6)	C_22_H_32_O_2_	351.230 (Na^+^)	351.239	9
		367.204 (K^+^)	367.201	−3
FA(22:5)	C_22_H_34_O_2_	369.220 (K^+^)	369.210	−10
LPC(14:0)	C_22_H_46_NO_7_P	468.309 (H^+^)	468.313	4
	C_22_H_44_NO_6_P	450.302 (H^+^, -H_2_O)	450.299	−4
LPC(15:0)	C_23_H_48_NO_7_P	482.325 (H^+^)	482.333	8
	C_23_H_46_NO_6_P	464.314 (H^+^, -H_2_O)	464.329	15
LPC(16:1)	C_24_H_48_NO_7_P	494.325 (H^+^)	494.329	4
		266.644 (H+K)^2+^	266.639	−5
	C_24_H_46_NO_6_P	476.314 (H^+^, -H_2_O)	476.312	−2
LPC(16:0)	C_24_H_50_NO_7_P	496.340 (H^+^)	496.342	2
		518.322 (Na^+^)	518.329	7
		267.652 (H+K)^2+^	267.64	−5
		478.330 (H^+^, -H_2_O)	478.330	0
		258.647 (H+K)^2+^	258.641	−6
LPC(18:4)	C_26_H_4_6NO_7_P	516.309 (H^+^)	516.315	6
LPC(18:3)	C_26_H_48_NO_7_P	518.329 (H^+^)	518.325	−4
	C_26_H_46_NO_6_P	500.314 (H^+^, -H_2_O)	500.301	−13
LPC(18:2)	C_26_H_50_NO_7_P	520.340 (H^+^)	520.339	−1
	C_26_H_48_NO_6_P	502.330 (H^+^, -H_2_O)	502.333	3
LPC(O-18:1)	C_26_H_54_NO_6_P	508.377 (H^+^)	508.380	3
		273.670 (H+K)^2+^	273.663	−7
LPC(18:1)	C_26_H_52_NO_7_P	522.356 (H^+^)	522.353	−3
		280.660 (H+K)^2+^	280.656	−4
	C_26_H_46_NO_6_P	504.345 (H^+^, -H_2_O)	504.347	2
LPC(18:0)	C_26_H_54_NO_7_P	524.372 (H^+^)	524.381	9
	C_26_H_52_NO_6_P	506.361 (H^+^, -H_2_O)	506.360	−1
LPC(20:5)	C_28_H_48_NO_7_P	542.325 (H^+^)	542.327	2
		290.644 (H+K)^2+^	290.648	4
		524.314 (H^+^, -H_2_O)	524.320	6
LPC(20:4)	C_28_H_50_NO_7_P	544.340 (H^+^)	544.349	9
	C_28_H_48_NO_6_P	526.330 (H^+^, -H_2_O)	526.324	−6
LPC(22:6)	C_30_H_50_NO_7_P	568.340 (H^+^)	568.348	8
		303.652 (H+K)^2+^	303.652	0
		550.349 (H^+^, -H_2_O)	550.349	0
PA(O-37:1)	C_40_H_79_O_7_P	703.564 (H^+^)	703.575	11
PC(16:0/14:1)*	C_38_H_74_NO_8_P	704.523 (H^+^)	704.524	1
		371.743 (H+K)^2+^	371.747	4
PC(16:0/14:0)*	C_38_H_74_NO_8_P	706.530 (H^+^)	706.541	11
PC(31:2) or PE(34:2)	C_39_H_74_NO_8_P	716.523 (H^+^)	716.538	15
PC(O-32:1) or PE(O-35:1)	C_40_H_80_NO_7_P	718.566 (H^+^)	718.567	1
PC (16:0/15:0)	C_39_H_78_NO_8_P	720.554 (H^+^)	720.557	3
PE(O-36:6)	C_41_H_72_NO_7_P	722.513 (H^+^)	722.512	−1
PC(32:2) or PE(35:2)	C_40_H_76_NO_8_P	730.538 (H^+^)	730.544	6
PC(16:0/16:1)*	C_40_H_78_NO_8_P	732.554 (H^+^)	732.553	−1
		385.759 (H+K)^2+^	385.752	−7
PC(33:5) or PE(36:5)	C_39_H_74_NO_9_P	738.507 (H^+^)	738.522	15
PC(O-34:2))	C_42_H_82_NO_7_P	744.591 (H^+^)	744.587	−4
PC(O-34:1)	C_42_H_84_NO_7_P	746.606 (H^+^)	746.593	−13
PS(O-34:1)	C_40_H_78_NO_9_P	748.541 (H^+^)	748.542	1
PC(34:4) or PE(37:4)	C_42_H_76_NO_8_P	754.531 (H^+^)	754.536	5
PC(16:0/18:3)*	C_42_H_78_NO_8_P	756.554 (H^+^)	756.557	3
PC(16:0/18:2)*	C_42_H_80_NO_8_P	758.570 (H^+^)	758.570	0
PC(16:0/18:1)*	C_42_H_82_NO_8_P	760.586 (H^+^)	760.582	−4
		399.775 (H+K)^2+^	399.778	3
PC(16:0/18:0)*	C_42_H_84_NO_8_P	762.601 (H^+^)	762.592	−9
PS(34:0) or	C_40_H_78_NO_10_P	764.544 (H^+^)	764.552	8
PC(O-36:6)	C_44_H_78_NO_7_P	764.559 (H^+^)	764.552	−7
PC(35:5)	C_43_H_76_NO_8_P	766.538 (H^+^)	766.555	17
PC(35:4)	C_43_H_78_NO_8_P	768.554 (H^+^)	768.561	7
PC(16:0/20:6)*	C_44_H_76_NO_8_P	778.539 (H^+^)	778.547	8
PC(16:0/20:5)*	C_44_H_78_NO_8_P	780.553 (H^+^)	780.551	−2
		409.759 (H+K)^2+^	409.760	1
PC(16:0/20:4)*	C_44_H_80_NO_8_P	782.570 (H^+^)	782.572	2
PC(16:0/20:3)*	C_44_H_82_NO_8_P	784.586 (H^+^)	784.588	2
PC(16:0/20:2)*	C_44_H_84_NO_8_P	786.601 (H^+^)	786.596	−5
PS(36:1) or	C_42_H_80_NO_10_P	790.560 (H^+^)	790.568	8
PC(O-38:7)	C_46_H_80_NO_7_P	790.575 (H^+^)	790.568	−7
PS(36:0)	C_42_H_82_NO_10_P	792.576 (H^+^)	792.584	8
PC(37:5) or PE(40:5)	C_45_H_80_NO_8_P	794.570 (H^+^)	794.5828	13
PS(O-38:5)	C_44_H_78_NO_9_P	796.549 (H^+^)	796.551	2
PC(38:8)	C_46_H_76_NO_8_P	802.5387 (H^+^)	802.547	8
PC(38:7)	C_46_H_78_NO_8_P	804.554 (H^+^)	804.558	4
PC(16:0/22:6)*	C_46_H_80_NO_8_P	806.570 (H^+^)	806.569	−1
		422.767 (H+K)^2+^	422.762	−5
PC(16:0/22:5)*	C_46_H_80_NO_8_P	808.586 (H^+^)	808.587	1
PS(38:4)	C_44_H_78_NO_10_P	812.544 (H^+^)	812.551	7
PS(38:1)	C_44_H_84_NO_10_P	818.591 (H^+^)	818.595	4
PC(39:6) or PE(42:6)	C_47_H_82_NO_8_P	820.586 (H^+^)	820.584	−2
PS(O-40:6)	C_46_H_80_NO_9_P	822.565 (H^+^)	822.574	9
PS(O-40:5)	C_46_H_82_NO_9_P	824.580 (H^+^)	824.578	−2
PC(40:9)	C_48_H_78_NO_8_P	828.554 (H^+^)	828.550	−4
PC(40:7)	C_48_H_82_NO_8_P	832.586 (H^+^)	832.585	−1
PS(40:5)	C_46_H_80_NO_10_P	838.560 (H^+^)	838.566	6
PS(40:4)	C_46_H_82_NO_10_P	840.575 (H^+^)	840.584	9
PS(38:1)	C_44_H_84_NO_10_P	840.573 (Na^+^)	840.584	11
PG(42:9)	C_48_H_77_O_10_P	845.533(H^+^)	845.529	−4
PG(40:6)	C_46_H_79_O_10_P	845.531 (Na^+^)	845.529	−2
PC(42:10)	C_50_H_80_NO_8_P	854.570 (H^+^)	854.561	−9
TAG(54:8)	C_57_H_94_O_6_	875.713 (H^+^)	875.710	−3
TAG(52:5)	C_55_H_96_O_6_	875.711 (Na^+^)	875.710	−1
TAG(54:7)	C_57_H_96_O_6_	877.729 (H^+^)	877.725	−4
TAG(52:4)	C_55_H_98_O_6_	877.726 (Na^+^)	877.725	−1
TAG(54:6)	C_57_H_98_O_6_	879.744 (H^+^)	879.740	−4
		901.726(Na^+^)	901.727	1
TAG(52:3)	C_55_H_100_O_6_	879.742 (Na^+^)	879.740	−2
TAG(54:5)	C_57_H_100_O_6_	881.760 (H^+^)	881.755	−5
		903.749 (Na^+^)	903.741	−8
TAG(52:2)	C_55_H_102_O_6_	881.757 (Na^+^)	881.755	−2
TAG(54:4)	C_57_H_102_O_6_	883.775 (H^+^)	883.771	−4
		905.757 (Na^+^)	905.756	−1
TAG(52:1)	C_55_H_104_O_6_	883.773 (Na^+^)	883.771	−2
PC(44:4)	C_52_H_96_NO_8_P	894.695 (H^+^)	894.708	13
PC(44:3)	C_52_H_98_NO_8_P	896.711 (H^+^)	896.721	10
		918.693 (Na^+^)	918.702	9
PC(42:0)	C_50_H_100_NO_8_P	896.708 (Na^+^)	896.721	13
PC(44:2)	C_52_H_100_NO_8_P	898.726 (H^+^)	898.733	7
PC(44:1)	C_52_H_102_NO_8_P	900.742 (H^+^)	900.741	−1
TAG(56:9)	C_59_H_96_O_6_	901.729 (H^+^)	901.727	−2
TAG(56:8)	C_59_H_98_O_6_	903.744 (H^+^)	903.741	−3
		925.726 (Na^+^)	925.739	13
TAG(56:7)	C_59_H_100_O_6_	905.760 (H^+^)	905.754	−6
		927.742 (Na^+^)	927.741	−1
TAG(56:6)	C_59_H_102_O_6_	907.776 (H^+^)	907.771	−5
		929.757 (Na^+^)	929.754	−4
TAG(54:3)	C_57_H_104_O_6_	907.773 (Na^+^)	907.772	−2
TAG(56:5)	C_59_H_104_O_6_	909.791 (H^+^)	909.788	−3
TAG(54:2)	C_57_H_106_O_6_	909.789 (Na^+^)	909.788	−1
PC(46:6)	C_54_H_96_NO_8_P	918.695 (H^+^)	918.702	7
TAG(58:11)	C_61_H_96_O_6_	925.727 (H^+^)	925.739	12
TAG(58:10)	C_61_H_98_O_6_	927.744 (H^+^)	927.741	−3
		949.726 (Na^+^)	949.737	11
TAG(58:9)	C_61_H_100_O_6_	929.760 (H^+^)	929.754	−6
		951.749 (Na^+^)	951.749	0
PC(46:5)	C_50_H_97_NO_13_	942.686 (Na^+^)	942.697	11
PC(46:3)	C_54_H_102_NO_8_P	946.724 (Na^+^)	946.726	2
TAG(60:13)	C_63_H_96_O_6_	949.729 (H^+^)	949.737	8
		971.711 (Na^+^)	971.714	3
TAG(60:12)	C_63_H_98_O_6_	951.744 (H^+^)	951.749	5
TAG(60:11)	C_63_H_100_O_6_	953.760 (H^+^)	953.757	−3
		975.742 (Na^+^)	975.754	12
TAG(58:8)	C_61_H_102_O_6_	953.757 (Na^+^)	953.757	0
TAG(60:10)	C_63_H_102_O_6_	955.776 (H^+^)	955.765	−11
TAG(58:7)	C_61_H_104_O_6_	955.773 (Na^+^)	955.765	−8
TAG(59:13)	C_62_H_94_O_6_	957.695 (Na^+^)	957.688	−7
TAG(62:16)	C_65_H_94_O_6_	971.713 (H^+^)	971.714	1
TAG(62:14)	C_65_H_98_O_6_	975.744 (H^+^)	975.754	10

*Lipids assignment based on tandem mass spectrometry.

### Animal Pole vs. Vegetal Pole

The unfertilized *X. laevis* egg is polarized, with a highly pigmented animal pole and a weakly pigmented vegetal pole separated by an unpigmented equatorial zone. During oogenesis, mRNAs and proteins are synthesized and differentially stored in the cytoplasm of these regions for use by the embryo after fertilization. In particular, mRNAs that direct ectodermal development are enriched in the animal pole whereas mRNAs that direct endoderm and gamete development are enriched in the vegetal pole [40], [41]. This molecular asymmetry is altered after fertilization by a cytoplasmic reorganization that occurs in response to sperm entry. This reorganization is critical for establishing the dorsal axis of the embryo. Because the mRNAs in the animal versus vegetal regions of the egg have very different developmental functions that are essential to establishing the vertebrate body plan, the corresponding metabolic and lipid profiles were investigated by LAESI-MS.

**Figure 6:**
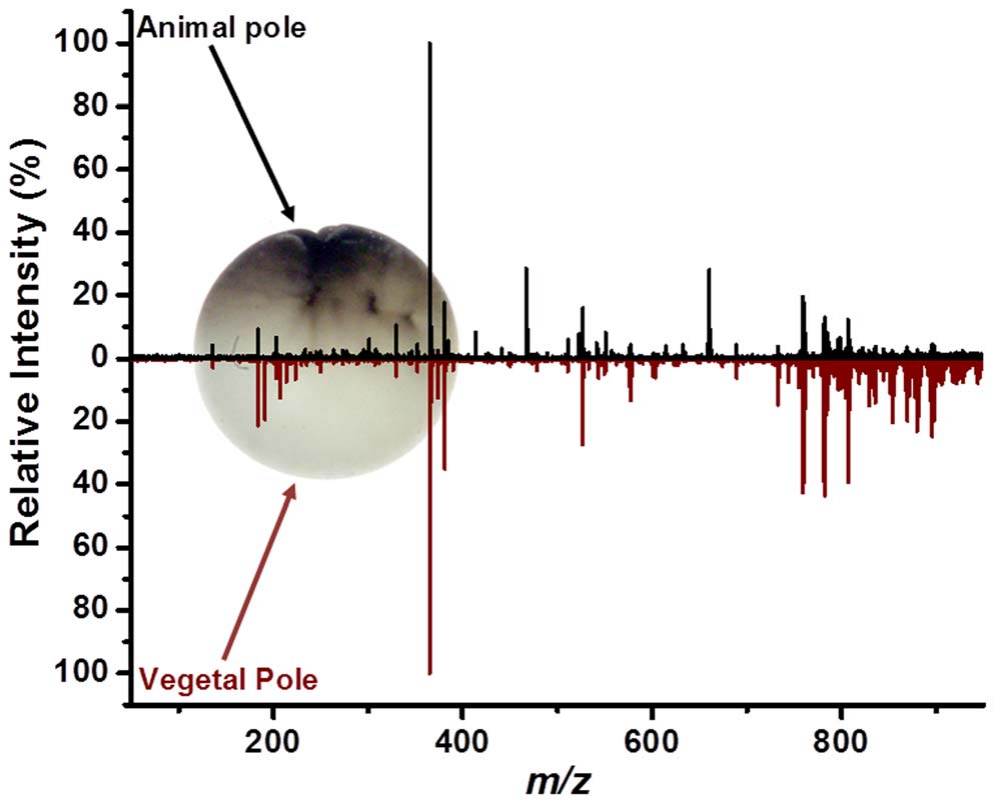
Comparison of positive ion LAESI mass spectra of animal and vegetal poles for unfertilized *X. laevis* ovum. The vegetal pole shows increased abundance of lipids relative to the animal pole. Average diameter of the egg in the overlaid image is ∼1.4 mm.

As seen in Fig. 6, numerous lipids were found in higher abundance at the vegetal pole compared to the animal pole. This finding was consistent with magnetic resonance imaging studies of the animal and vegetal poles in *X. laevis* oocytes that had shown two to three times higher abundance for TAGs in the vegetal region [42]. Among the many lipids detected at the vegetal pole, we found an LPC at *m/z* 478, which was consistent with the presence of yolk platelets there. A previous report suggested that LPC lipids played a role in assisting fertilization [12]. In addition, there were many small metabolites found at both poles and their putative identifications can be found in Table 1. Although differences in the small metabolite abundances at the two poles were clearly discernable, they exhibited significant variance between eggs. As an example, the ion abundance ratios between vegetal and animal poles are shown in Table 3 for certain metabolites and lipids. To establish reliable abundance ratios, further analysis is needed.

**Table 3:** Metabolite and lipid ion abundance ratios for vegetal and animal poles of *X. laevis* egg.

Metabolite or Lipid	Ion	*m/z*	Abundance ratio vegetal/animal	Abundance ratio animal/vegetal
choline	[+]	104.1	3.7	0.3
adenine	[H^+^]	136.1	1.0	1.0
guanine or hydroxyadenine	[Na^+^]	174.0	4.9	0.2
arginine	[H^+^]	175.1	1.8	0.6
phosphocholine	[H^+^]	184.1	5.2	0.2
monosaccharide	[Na^+^]	203.1	1.2	0.9
acetyldihydrolipoamide	[H^+^]	250.1	2.8	0.4
glutathione	[Na^+^]	330.1	0.6	1.7
glutathione	[K^+^]	346.0	0.7	1.5
disaccharide or trehalose	[Na^+^]	365.1	1.9	0.5
disaccharide or trehalose	[K^+^]	381.1	2.8	0.4
unknown *m/z* 374.033	-	374.0	7.5	0.1
cholesterol	[H^+^-H_2_O]	369.4	4.7	0.2
adenosine diphosphate (ADP)	[H^+^]	428.0	0.5	1.9
adenosine diphosphate (ADP)	[Na^+^]	450.0	1.7	0.6
unknown *m/z* 467.101	-	467.1	0.1	17.0
LPC(16:1)	[H^+^-H_2_O]	476.3	4.1	0.2
LPC(16:0)	[H^+^-H_2_O]	478.3	4.8	0.2
adenosine triphosphate (ATP)	[H^+^]	508.0	1.4	0.7
adenosine triphosphate (ATP)	[Na^+^]	530.0	0.6	1.8
unknown *m/z* 522.590	-	522.6	1.3	0.7
trisaccharides	[Na^+^]	527.2	2.4	0.4
unknown *m/z* 541.126	-	541.1	1.2	0.8
Cer(d 40:1)	[Na^+^]	660.6	0.1	11.6
unknown *m/z* 689.201	-	689.2	2.0	0.5
PC(16:0/16:1)	[H^+^]	732.6	5.6	0.2
PC(34:4) or PE(37:4)	[H^+^]	754.5	9.2	0.1
PC(16:0/18:3)	[H^+^]	756.6	3.9	0.3
PC(16:0/18:2)	[H^+^]	758.6	3.7	0.3
PC(16:0/18:1)	[H^+^]	760.6	4.3	0.2
PC(16:0/18:0)	[H^+^]	762.6	3.3	0.3
PC(16:0/20:5)	[H^+^]	780.6	5.9	0.2
PC(16:0/20:4)	[H^+^]	782.6	6.6	0.2
PC(16:0/20:3)	[H^+^]	784.6	3.7	0.3
PS(O-38:5)	[H^+^]	796.5	3.0	0.3
PC(16:0/22:6)	[H^+^]	806.6	5.8	0.2
PC(16:0/22:5)	[H^+^]	808.6	5.5	0.2
TAG(54:6)+[H+] or	[H^+^] or	879.7	18.0	0.1
TAG(52:3)+[Na+]	[Na^+^]	879.7	18.0	0.1
TAG(52:3)	[K^+^]	895.7	9.9	0.1
TAG(52:2)	[K^+^]	897.7	9.4	0.1
TAG(56:9)	[H^+^]	901.7	9.2	0.1
TAG(56:8)	[H^+^]	903.7	16.8	0.1
TAG(54:6)	[K^+^]	917.7	9.8	0.1
TAG(54:5)	[K^+^]	919.7	7.3	0.1
TAG(54:4)	[K^+^]	921.7	6.7	0.1
TAG(60:13)	[H^+^]	949.7	7.3	0.1

### Fertilized Ovum and Early Embryos

A number of critical developmental events occur after the *X. laevis* egg is fertilized, including: sperm nucleus entry into the cytoplasm and an influx of calcium, the completion of meiosis II, the initiation of synchronous cell cycles, and the initiation of low levels of gene transcription at the 32-cell stage [37]. In the unfertilized egg, the cell cycle is arrested in metaphase of the second meiotic division, but all of the components for cell division, cytokinesis, gene transcription and protein synthesis have previously been produced in the oocyte.

**Figure 7:**
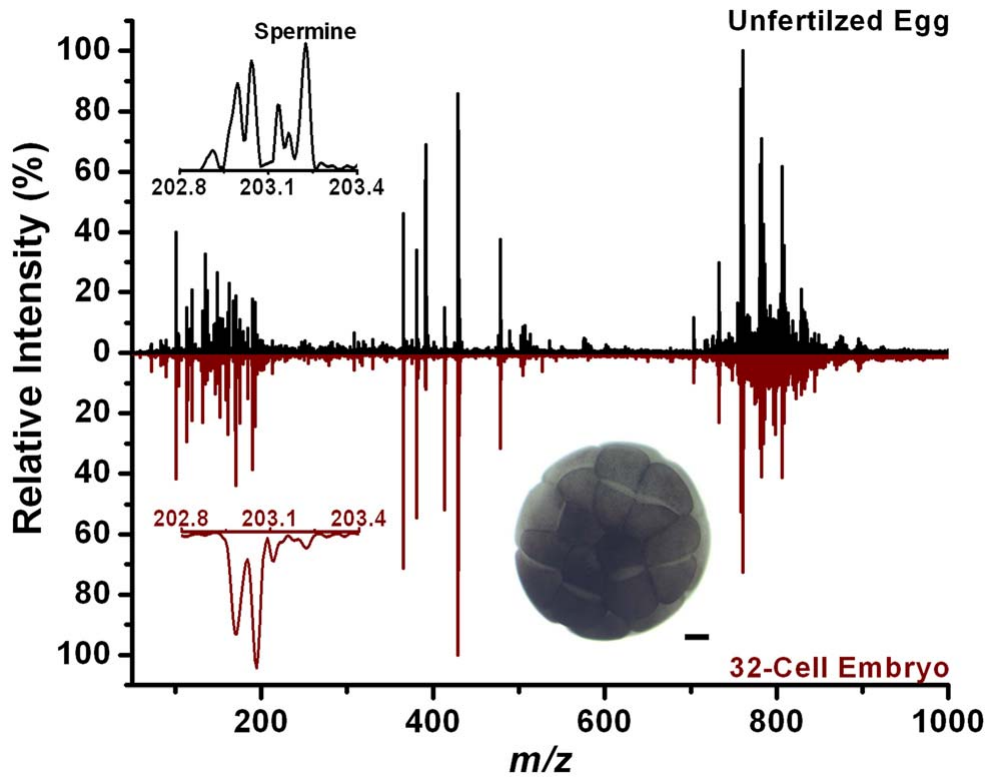
Positive ion LAESI mass spectra of an unfertilized *Xenopus* ovum and a 32-cell embryo show a very similar metabolite profiles with some key differences, e.g., the absence of spermine in the embryo spectrum. The inset shows a *Xenopus* embryo at the 32-cell stage. The scale bar in the inset is 200 µm.

We directly analyzed unperturbed eggs by LAESI-MS at 30 minute intervals after fertilization, and cleavage stage embryos at 8-cells and 32-cells. Most of the major metabolites and lipids found during these early stages after fertilization were similar to those in unfertilized *Xenopus* eggs (see Fig. 7). These results are consistent with the prevailing concept that the synthesis of the major cellular components occurs during oogenesis. However, some subtle differences between the some key metabolites were observed in the spectra. Most notably, spermine was consistently detected in the unfertilized eggs, but was absent in the 32-cell stage embryos. It is well documented that the concentration of spermine decreases during oogenesis leading to the reduction of total polyamines required for maturation [43], [44].

In both jellied and dejellied systems, the intensities for the following ions from stage 8 embryos were consistently lower than from unfertilized eggs: *m/z* 113.884, 114.892, 150.009, 175.118 (arginine), 199.991, 231.934, 239.919 and 277.199. Conversely, the following ions showed higher abundances in stage 8 embryos: 113.104, 131.111 (acetylputrescine), 157.127, 261.075, 239.101, 277.054, 283.066, 299.038, 315.015, 239.236, 241.098, 284.066, 295.021, 542.052 and 660.618 (Cer(d18:0/23:0)). Furthermore, there is a higher abundance of most TAGs in stage 8 embryos compared to unfertilized dejellied *Xenopus* eggs.

## Conclusions

There has been extensive of the transcriptome that regulates the major developmental transitions in *X. laevis* at the subcellular level. These investigations have been limited to the detection of mRNAs by *in situ* hybridization and RT-PCR approaches and to the detection of proteins by immunohistochemistry or Western blotting techniques. The latter have been hampered by the lack of antibodies against proteins of interest. In addition, these approaches reliably detect mRNAs and proteins that are in high abundance. Metabolites and lipids have not been studied in detail at in single eggs because of the lack of appropriately sensitive technologies that can assess these molecules in the living egg/embryo in its natural state. Therefore, evaluation of eggs and embryos with LAESI-MS was performed to determine whether subtle changes in metabolites and lipids and their subcellular profiles are detectable.

In this report the direct atmospheric pressure analysis of *X. laevis* eggs and early cleavage stages embryos is presented. The results of the LAESI-MS metabolic and lipidomic profiles of the *Xenopus* eggs were compared to previous literature results and showed a wide coverage for lipids and small metabolites. Reactive-LAESI MS/MS, with in-plume cationization of PC lipids without sample preparation, was utilized to identify the acyl chains. An investigation of the animal and vegetal poles of the *X. laevis* ovum showed increased abundance of lipids in the vegetal pole relative to the animal pole. Radial profiling of a jelly coated egg by LAESI-MS utilizing consecutive laser pulses revealed dramatic compositional changes between the jelly coat the vitelline/plasma membranes and the cytoplasm.

Finally, LAESI-MS was used to demonstrate that subtle metabolic profile changes could be detected after the egg was fertilized and synchronous cell cycles were initiated. Of particular note, spermine was depleted after fertilization, which consistent with the reduction of total polyamines required for embryo maturation [43], [44]. Major differences between the metabolites of unfertilized egg and the early cleavage stage embryos were not expected because all the needed organelles and biochemical building blocks needed to carry the embryo through the first 8 hours of development had previously been synthesized and stored in the oocyte. Nonetheless, the sensitivity of LAESI-MS enabled the detection of a few subtle differences, and uncovered novel molecules that are asymmetrically distributed between the animal, ectoderm-forming, and vegetal, endoderm-forming, regions of the egg. This simple approach, partially validated here for a vertebrate embryo for which there is abundant traditional biochemical data, should be ideal for the direct analyses of other embryos that are rare and smaller in size.
